# Use of the Systematized Nomenclature of Medicine Clinical Terms (SNOMED CT) for Processing Free Text in Health Care: Systematic Scoping Review

**DOI:** 10.2196/24594

**Published:** 2021-01-26

**Authors:** Christophe Gaudet-Blavignac, Vasiliki Foufi, Mina Bjelogrlic, Christian Lovis

**Affiliations:** 1 Division of Medical Information Sciences Geneva University Hospitals Geneva Switzerland; 2 Department of Radiology and Medical Informatics University of Geneva Geneva Switzerland

**Keywords:** SNOMED CT, natural language processing, scoping review, terminology

## Abstract

**Background:**

Interoperability and secondary use of data is a challenge in health care. Specifically, the reuse of clinical free text remains an unresolved problem. The Systematized Nomenclature of Medicine Clinical Terms (SNOMED CT) has become the universal language of health care and presents characteristics of a natural language. Its use to represent clinical free text could constitute a solution to improve interoperability.

**Objective:**

Although the use of SNOMED and SNOMED CT has already been reviewed, its specific use in processing and representing unstructured data such as clinical free text has not. This review aims to better understand SNOMED CT's use for representing free text in medicine.

**Methods:**

A scoping review was performed on the topic by searching MEDLINE, Embase, and Web of Science for publications featuring free-text processing and SNOMED CT. A recursive reference review was conducted to broaden the scope of research. The review covered the type of processed data, the targeted language, the goal of the terminology binding, the method used and, when appropriate, the specific software used.

**Results:**

In total, 76 publications were selected for an extensive study. The language targeted by publications was 91% (n=69) English. The most frequent types of documents for which the terminology was used are complementary exam reports (n=18, 24%) and narrative notes (n=16, 21%). Mapping to SNOMED CT was the final goal of the research in 21% (n=16) of publications and a part of the final goal in 33% (n=25). The main objectives of mapping are information extraction (n=44, 39%), feature in a classification task (n=26, 23%), and data normalization (n=23, 20%). The method used was rule-based in 70% (n=53) of publications, hybrid in 11% (n=8), and machine learning in 5% (n=4). In total, 12 different software packages were used to map text to SNOMED CT concepts, the most frequent being Medtex, Mayo Clinic Vocabulary Server, and Medical Text Extraction Reasoning and Mapping System. Full terminology was used in 64% (n=49) of publications, whereas only a subset was used in 30% (n=23) of publications. Postcoordination was proposed in 17% (n=13) of publications, and only 5% (n=4) of publications specifically mentioned the use of the compositional grammar.

**Conclusions:**

SNOMED CT has been largely used to represent free-text data, most frequently with rule-based approaches, in English. However, currently, there is no easy solution for mapping free text to this terminology and to perform automatic postcoordination. Most solutions conceive SNOMED CT as a simple terminology rather than as a compositional bag of ontologies. Since 2012, the number of publications on this subject per year has decreased. However, the need for formal semantic representation of free text in health care is high, and automatic encoding into a compositional ontology could be a solution.

## Introduction

### Background

The ability to meaningfully exchange and process data is of utmost importance in health care, whether it is inside a hospital setting either among different health structures or among health systems in different countries [1-3]. The use of a common terminology is a way to improve both interoperability and the secondary use of data [4].

The Systematized Nomenclature of Medicine Clinical Terms (SNOMED CT) was created in 1999 by the fusion of 2 important health care terminologies—SNOMED reference terminology (SNOMED RT) and Clinical Terms Version 3. It was first released in 2002. SNOMED CT is currently considered as the most comprehensive, multilingual, clinical health care terminology in the world, with more than 350,000 concepts and a million relationships [5-7]. It is maintained and published by SNOMED International, a nonprofit organization comprising 39 member countries [8]. In the last 18 years, SNOMED CT has grown in size and coverage and has been included as a standard vocabulary in the meaningful use program [9]. This is an important step for any electronic health record willing to attain interoperability.

With 3 components, namely concepts, descriptions, and relationships, SNOMED CT can be observed as both a complex ontology and a graph containing vertices and labeled edges. This structure allows interesting features such as compositional grammar, expression constraint queries, or postcoordination. It is therefore possible to create postcoordinated concepts that represent new meanings not present in the terminology. These postcoordinated concepts can then be queried and processed with the rest of the terminology [5,10,11].

These characteristics, similar to those of a natural language, make SNOMED CT a candidate for representing clinical free text in a semantically rich, machine-readable manner. Although encoding free text into SNOMED CT can be done manually, it is costly and not scalable for large data sets. Therefore, it is often accomplished by natural language processing (NLP). NLP is an active research branch in the biomedical field and has been broadly applied in the scientific literature and clinical text for diverse tasks [12-14]. However, NLP applications on clinical documents are less frequent. Among the reasons explaining this disparity are the limited access to corpora of clinical documents and the lack of publicly available annotated corpora [15]. These barriers are even more important for languages other than English.

### Objectives

SNOMED CT has already been the subject of many studies and evaluations of its coverage, ability to represent complex concepts, or usability in a clinical setting [16-19]. Its usage has already been a subject of reviews; however, those publications are older than 10 years [13,20] or focus on its general use without focusing on its usage to process and represent unstructured data such as clinical free text [7]. Therefore, this work aims to better understand the use of SNOMED CT for representing free text in medicine via a scoping systematic review. It also aims to decipher the use of this terminology across fields, languages, and countries and how it is used from an analytical point of view, such as terminology source up to exploiting its advanced features, that is, postcoordination and compositional grammar.

## Methods

### Article Selection Process

An exploratory research performed using text-based queries on MEDLINE and Google Scholar helped in defining the queries, topics, and objectives of this study. This work led to the selection of 3 databases for the review based on previous reviews addressing similar topics [7,20,21]. This choice was made to increase coverage. Purely engineering-related databases, such as the Institute of Electrical and Electronics Engineers Xplore or the Association for Computing Machinery digital library, were not selected because of the technical content of their publications, which was often not related to real clinical settings.

In this work, clinical free text is considered as any text written in a natural language about a patient, which does not come from a finite value set. Free-text fields in structured forms and problem lists have been included to broaden the scope.

The selected databases were PubMed [22], Embase [23], and Web of Science [24]. The final query used was as follows: (“SNOMED-CT” OR “SNOMED CT”) AND (“free-text” OR “free text” OR “narrative”). These keywords were defined during the preliminary research. The bottleneck was the presence of the term “SNOMED CT,” and no other synonyms of narrative or free text were added as they did not change the results. The final query was made on August 9, 2019.

To be selected, an article must meet the following inclusion criteria:

- It should be published in scientific journals or conference proceedings after 2002.
- It should include the usage of SNOMED CT to represent or process clinical free text.

The limitation on the date was set to avoid publications that focused on the previous versions of SNOMED.

Although the selection was voluntarily broad, white papers, editor papers, posters, or conference abstracts were excluded. Articles not available in English were also excluded. The Unified Medical Language System (UMLS) [25] developed by the National Library of Medicine (NLM) combines biomedical terminologies in a single resource. Since the release of the UMLS-labeled 2004AA [26,27], it contains SNOMED CT. In this work, publications focusing on the usage of UMLS were included only if they specifically mentioned the usage of SNOMED CT.

To be as inclusive as possible on the chosen topic, the references in every publication were also reviewed to include new publications. The recursive reference review was stopped when no additional publications were added to the set. This has been done with the aim of reducing the impact of the query on the final selection of articles. Moreover, 3 review articles about information extraction from clinical free text were included in the selection. Despite not meeting the inclusion criteria, they were considered as a source of reference to other publications meeting the criteria. Obviously, they were not the target of the topic review described below.

### Topics Reviewed

The articles were then studied to extract some specific topics in a systematic manner. The first topic reviewed was the type of document used as a free-text source. To better detect which data were used in these publications, we defined the categories described in Textbox 1.

**Categories of documents.**

- History and physical examinations: this category includes documents summarizing the situation of a patient admitted in a health care structure, and his or her physical examination such as admission notes
- Clinical summaries: this category includes any document summarizing a care episode such as a discharge summary
- Death certificates
- Problem lists: this category regroups documents listing the problems of a patient admitted in a health care structure
- Autopsy reports
- Incident reports
- Allergy reports
- Complementary exam reports: this category regroups any document related to a complementary exam, including but not limited to radiology, pathology, and genomic reports
- Narrative notes: this category includes progress notes, nurse notes, and clinical notes not further specified
- Various: this category was selected when a publication used more than one type of document according to this classification

The publications were then classified according to the language they targeted in their work. All the selected publications included a part where the free text was mapped to SNOMED CT concepts. This terminology binding step was classified depending on its justification and whether it was the final goal of the research or a step toward another goal. Textbox 2 defines the types of reasons. These reasons have been defined empirically to fully cover the possibilities encountered in publications. For each type, a point was added if it was present in the publication.

The method used for the terminology binding to SNOMED CT was classified as “manual,” “rule-based,” “machine learning,” or “hybrid” for each article. The definitions used for these categories are listed in Textbox 3. When mapping was accomplished using a specific software, it was reviewed.

The general usage of SNOMED CT was reviewed on 2 specific topics: whether the full terminology or a subset of concepts was used and whether more advanced features of SNOMED CT were included in the study.

**Categories classifying the reason for the terminology binding to Systematized Nomenclature of Medicine Clinical Terms.**

- Information extraction: Systematized Nomenclature of Medicine Clinical Terms (SNOMED CT) is used to extract meaningful information from free text. The focus must be aimed at extracting information, not structuring or encoding it. Publications using the terminology binding to extract clinical information from documents that fall under this category
- Data normalization: SNOMED CT is used to encode existing data. This category is different from information extraction because it focuses on adding semantics to the data while keeping it intact. It includes publications where SNOMED CT is used to define a template or to support information entry
- Synonym resource: SNOMED CT includes synonyms for a large number of its concepts. In this category, SNOMED CT is used as a source for synonyms
- Quality evaluation: SNOMED CT is used to evaluate the quality of care or documentation
- Coverage evaluation: The focus is aimed at evaluating the coverage of SNOMED CT for a specific task by mapping it to free text
- Similarity evaluation: SNOMED CT is used to evaluate similarity among data. It is usually made by using the relationships present in SNOMED CT to compute the semantic distance between concepts
- Gold standard creation: SNOMED CT is used to create a gold standard data set
- Feature in a classification task: SNOMED CT mapping is used as a feature in a classification task
- Value set creation: SNOMED CT is used to define a specific value set
- Mapping to other terminologies: SNOMED CT is used as a bridge to other terminologies

**Definition of the categories used to classify the mapping method.**

- Manual: the mapping is made by manually reading the text and assigning the correct concept [28,29]
- Rule-based: the mapping is made using rule-based methods such as text search, regular expressions, finite state machines, or a tool that is defined as rule-based [30,31]
- Machine learning: the mapping is made using probabilistic algorithms based on a learning mechanism such as support vector machine, conditional random fields [32], or naïve Bayes [33]
- Hybrid: the mapping is made using both rule-based and machine learning methods, whether it is simultaneously combined or sequentially [34]

## Results

### Article Selection

**Figure 1:**
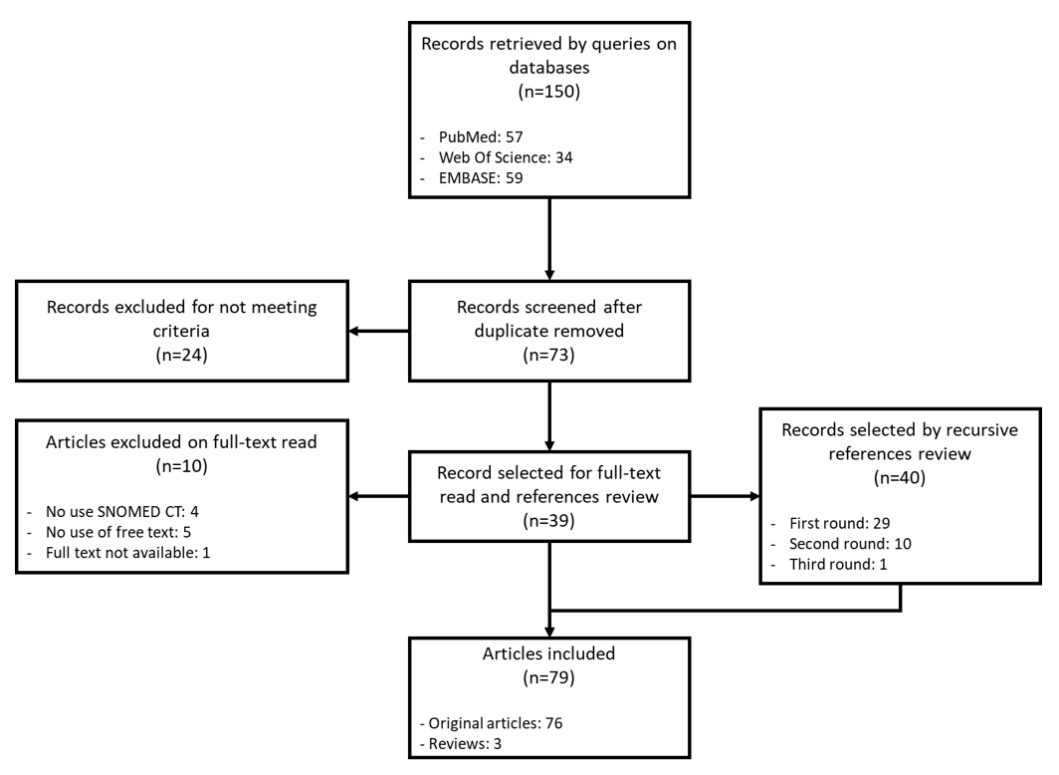
Flow diagram of the selection process. SNOMED CT: Systematized Nomenclature of Medicine Clinical Terms.

After 3 rounds of recursive reference review, the final selection included 76 publications and 3 reviews. Complete list of the publications is provided in Multimedia Appendix 1 [14,16,28-101]. Those reviews [13,102,103] will be excluded from the rest of the analysis, as they were only studied to broaden the scope of this review. The flow diagram according to PRISMA (Preferred Reporting Items for Systematic Reviews and Meta-Analyses) [104] is shown in Figure 1.

**Figure 2:**
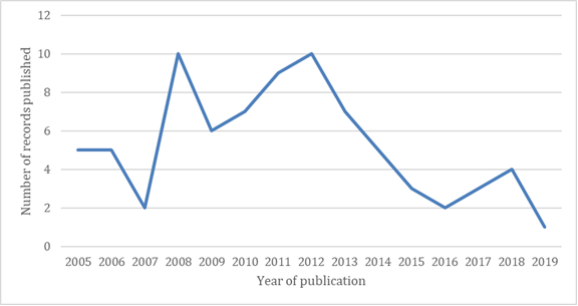
Number of publications per year of publication.

Among the 76 selected articles, 42 (55%) publications were journal articles and 34 (45%) were conference proceedings. The number of publications published per year is shown in Figure 2. The 76 publications were issued from 37 journals and conference proceedings, with 10 journals or proceedings appearing in more than one publication in the selection (Table 1).

**Figure 3:**
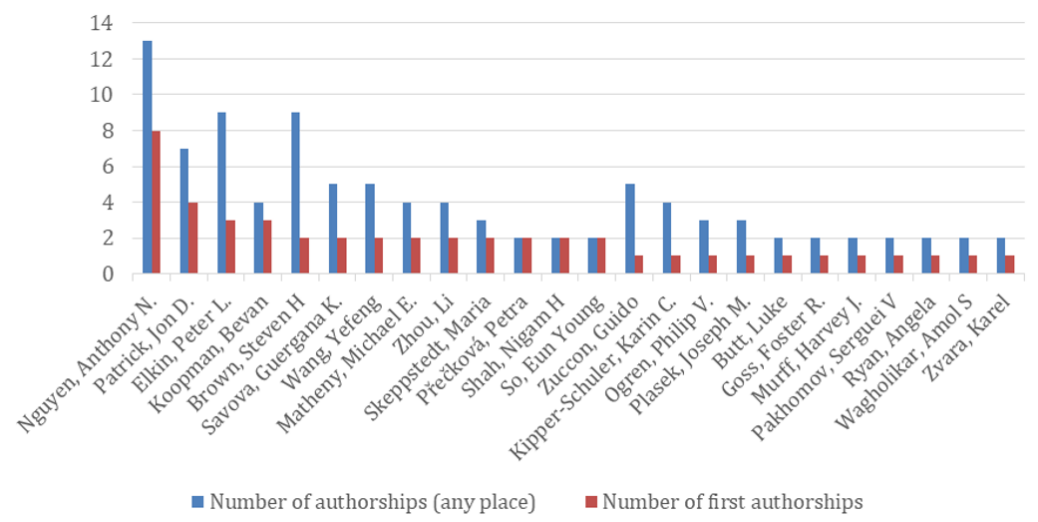
Number of authorships for the most prolific authors in selection.

Overall, 238 unique authors were credited in the selection. More prolific authors (more than one authorship) are displayed in Figure 3.

**Table 1:** Journals and conferences having more than 1 article in the selection.

Name of journal or conference	Publications, n
*AMIA^a^* *Annual Symposium proceedings*	15
*Journal of Biomedical Informatics*	8
*BMC^b^* *Medical Informatics and Decision Making*	7
*Journal of the American Medical Informatics Association*	7
*Studies in Health Technology and Informatics*	3
*Journal of Digital Imaging*	2
*AMIA Joint Summits on Translational Science proceedings*	2
*Mayo Clinic Proceedings*	2
*Electronic Journal of Health Informatics*	2
*International Journal of Medical Informatics*	2

^a^AMIA: American Medical Informatics Association.

^b^BMC: BioMed Central.

### Type of Data

The types of documents used in each publication are summarized in Table 2. The most frequent types are complementary exam reports (18/76, 24%), followed by narrative notes (16/76, 21%) and publications using more than one type of document (14/76, 18%).

**Table 2:** Number of publications per type of document used for the mapping.

Document Type	Publications (N=76), n (%)
Complementary exam report	18 (24)
Narrative note	16 (21)
Various	14 (18)
History and physical examination	8 (11)
Clinical summary	6 (8)
Death certificate	5 (7)
Problem list	3 (4)
Not available	3 (4)
Incident report	1 (1)
Autopsy report	1 (1)
Allergy report	1 (1)

### Language

The target languages in the publications are listed in Table 3. Most papers focused on English (69/76, 91%). The 3 other languages were Swedish, Czech, and Chinese (Table 3).

**Table 3:** Target language in publications.

Language	Publications (N=76), n (%)
English	69 (91)
Swedish	3 (4)
Czech	3 (4)
Chinese	1 (1)

### Reason for the Terminology Binding to SNOMED CT

As the focus of this work is to depict how the research community uses SNOMED CT to process clinical free text, selected articles had to include a part in which free-text data were mapped to SNOMED CT concepts. However, the mapping part was only a step toward another goal in many cases (eg, classification task [35,36], similarity measures [29,37], etc; Table 4).

**Table 4:** Role of the Systematized Nomenclature of Medicine Clinical Terms mapping in the publications.

Role of the SNOMED CT^a^ mapping	Publications (N=76), n (%)
Final goal	16 (21)
Part of final goal	25 (33)
Step toward other goal	35 (46)

^a^SNOMED CT: Systematized Nomenclature of Medicine Clinical Terms.

The reasons for the SNOMED CT mapping in publications are displayed in Table 5. The most frequent reason is information extraction (44/76, 39%), followed by feature in a classification task (26/76, 23%) and data normalization (23/76, 20%). The remaining categories appear in 5 publications or less.

**Table 5:** Reason for the mapping in publications.

Reason for the SNOMED CT^a^ mapping	Publications, n (%)
Information extraction	44 (39)
Feature in a classification task	26 (23)
Data normalization	23 (20)
Coverage evaluation	5 (4)
Similarity evaluation	4 (4)
Quality evaluation	3 (3)
Value set creation	3 (3)
Synonym resource	2 (2)
Terminology mapping	2 (2)
Gold standard creation	1 (1)
Total number of points given	113 (100)

^a^SNOMED CT: Systematized Nomenclature of Medicine Clinical Terms.

### Mapping Method

**Figure 4:**
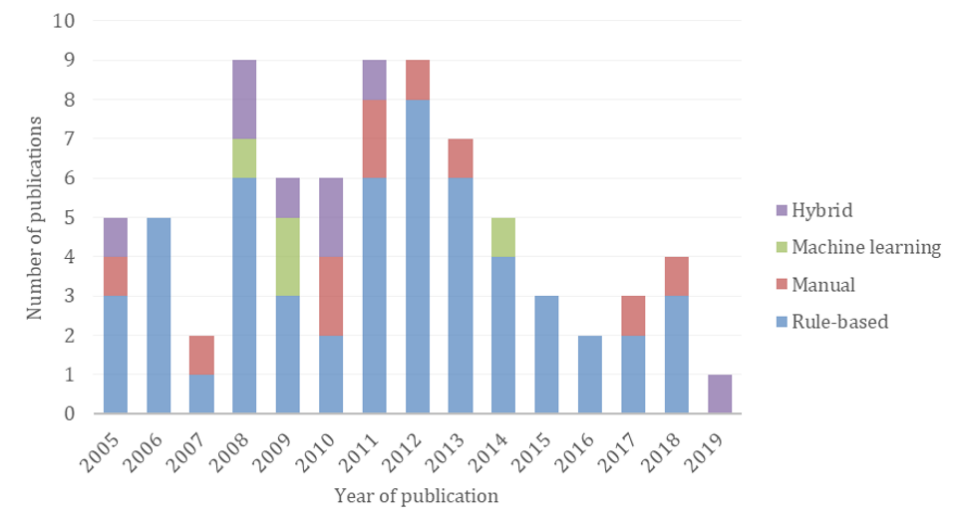
Number of articles applying a specific method for Systematized Nomenclature of Medicine Clinical Terms (SNOMED CT) mapping when available.

The type of method used for mapping according to the previously defined classification is presented in Table 6, and the methods used per year is displayed in Figure 4. The evolution of the methods shows that articles presenting machine learning approaches were published only in 2008, 2009, and 2014. Hybrid approaches are present during the period 2005 to 2010 and in 2019. With 70% (53/76) of publications, rule-based approaches were the most common method used to perform this task, although the number of publications per year is reducing overall.

**Table 6:** Method used for mapping free-text data to Systematized Nomenclature of Medicine Clinical Terms.

Method for SNOMED CT^a^ mapping	Publications (N=76), n (%)
Rule-based	53 (70)
Manual	11 (14)
Hybrid	8 (11)
Machine learning	4 (5)

^a^SNOMED CT: Systematized Nomenclature of Medicine Clinical Terms.

### Software Used for Terminology Binding

Table 7 shows the software used to specifically map free-text data to SNOMED CT concepts, the number of publications in which they appear, and whether they are publicly available. Only software used to produce a mapping into SNOMED CT are considered. Software used only for a step of the NLP pipeline such as negation detection or tokenization and not resulting in a concept-mapping output are not listed.

**Table 7:** Tools used for mapping free text to Systematized Nomenclature of Medicine Clinical Terms concepts.

Name of tool	Publications, n	Availability for public use
Medtex	12	No
MCVS^a^	8	No
MTERMS^b^	4	No
MetaMap	3	Yes
MetaMap transfer	3	Yes
Open biomedical annotator	2	Yes
MedLEE^c^	2	No
cTAKES^d^	2	Yes
Lingoengine	1	Yes
Snapper	1	No
iSCOUT	1	No
RapTAT^e^	1	No

^a^MCVS: Mayo Clinic Vocabulary Server.

^b^MTERMS: Medical Text Extraction Reasoning and Mapping System.

^c^MedLEE: Medical Language Extraction and Encoding System.

^d^cTAKES: clinical Text Analysis and Knowledge Extraction System.

^e^RapTAT: Rapid Text Annotation Tool.

Although all the software aim to detect concepts in free text, the wide disparities in methods and evaluation metrics, the subsets of concepts used, and the output terminologies prevent strict comparison. Therefore, the following review focuses only on the systems themselves and their published evaluation.

*Medtex* [38]**,** developed by the Australian eHealth research center, is built based on other existing tools (GATE [105], metamap transfer MMTx [106], and NegEx [107]) and can annotate free text with SNOMED CT concepts and negation marks. Although it is used in 12 publications, to the best of our knowledge, no strict evaluation of the mapping has been published.

The *Mayo Clinic Vocabulary Server* (MCVS) [16], also called Multi-threaded Clinical Vocabulary Server [39], is able to map free text to codes in various classifications, among which, SNOMED CT codes. It is the subject of an evaluation of over 4996 problem statements, which resulted in a sensitivity of 99.7% and a specificity of 97.9%. It is linked to *LingoEngine* [40], which is described as a commercially available product linked to MCVS.

The *Medical Text Extraction Reasoning and Mapping System (MTERMS)* [41] is a system that uses shallow and deep parsers to extract and structure information from free text by using local and standard terminologies. The system also proposes mappings between the terminologies. It has been used to extract medication information, allergens, allergic reactions, [42,43] and family relatives [44]. Each of these uses required specific customization, such as adding ad hoc dictionaries. Evaluations proposed in publications about MTERMS cover the encoding of information in multiple terminologies and are restricted to a specific subject. The evaluation of allergy data shows a precision of 84.4%, a recall of 91.0%, and an F-measure of 87.6%. Moreover, the evaluation of family relatives showed a precision of 100%, a recall of 97.4%, and an F-measure of 98.7% over 291 occurrences.

*MetaMap* [106]**,** and its Java implementation *MMTx*, was developed by the NLM. Its goal is to map the biomedical text to the UMLS Metathesaurus [108]. Since 2004, the UMLS Metathesaurus contains SNOMED CT. Although MetaMap only maps free text to the UMLS concept unique identifier(CUI), the link between a CUI and a SNOMED CT concept is present in the Metathesaurus and it is possible to specify vocabulary sources used for mapping. Therefore, in this work, MetaMap is considered as a tool that can map free text to SNOMED CT concepts. A realistic evaluation of the performance of this software has never been performed [109]. However, specific task evaluations and comparisons with other software have been published [110-112]. They showed a performance of 88% in recall, 89% in precision, and 88% in F-score on clinical notes; a precision of 85% and a recall of 78% on concepts extracted from medical curriculum documents [110]; and finally, a precision between 33% and 76% on multiple web-based biomedical resources for the mapping of biological processes, depending on the data sources [111]. However, no specific evaluation of the SNOMED CT mapping has been published.

The *Open Biomedical Annotator* (*OBA*) [113] is an ontology-based web service that can annotate free text with a variety of ontologies. It uses and improves the annotations of a concept recognizer called Mgrep [114] and is developed by the National Center for Integrative Biomedical Informatics at the University of Michigan. Publications using OBA in the selection did not propose an evaluation of the SNOMED CT mapping. However, a comparison of Mgrep with Metamap showed a precision between 58% and 93% for biological processes depending on the data source [111]. However, these evaluations are not focused on SNOMED CT.

The *Medical Language Extraction and Encoding System* [115] developed in Columbia University aims to transform clinical data into controlled vocabularies. It has been specifically adapted for UMLS and evaluated on 300 random sentences with a precision of 89% and a recall of 83% [116]. However, this evaluation does not mention SNOMED CT or the UMLS version used.

The *clinical Text Analysis and Knowledge Extraction System* (*cTAKES*) [45], developed in the Mayo Clinic, is an open-source NLP software aimed at information extraction. It includes a dictionary lookup component able to map the free-text data to UMLS concepts. The named entity recognition component has been evaluated on a corpus of 160 notes manually annotated with UMLS concepts including SNOMED CT, and shows an F-score of 71.5% for exact and 82.4% for overlapping spans [46].

*Snapper* [117] by the Australian eHealth research center is a software with the ability to input free-text data and perform the mapping from a terminology to SNOMED CT. To the best of our knowledge, no strict evaluation of the software has been performed. *Snapper* has been used in the selection to classify narratives into symptom groups [47].

*ISCOUT* appears in only one publication in the selection. This software, developed at the Brigham and Women's Hospital in Boston, is used internally for document retrieval according to a list of terms from a terminology [48]. In the publication, it is used with a list of concepts from various terminologies, including SNOMED CT, to retrieve documents. However, no evaluation of concept detection is proposed.

The *Rapid Text Annotation Tool* (*RapTAT*) [33] is a token order–specific naïve Bayes–based machine learning system designed to predict an association between phrases and concepts. It has been evaluated on the manually annotated 2010 i2b2 shared task data [118] and compared with the MCVS output, defined as the gold standard on 2860 discharge summaries. On the manual data set, *RapTAT* reached a precision of 95%, a recall of 96%, and an F-measure of 95%. To reproduce the MCVS output, *RapTAT* achieved a precision of 92%, a recall of 85%, and an F-measure of 89%.

Among all software, 5 are available, either as a web-based interface or as an installer for public usage. For example, Metamap, MMTx, and cTAKES are open source, OBA is available as a web-based interface, and LingoEngine is commercially available.

### Subset Usage

As SNOMED CT includes more than 340,000 concepts, the research studies described in publications often restrict their usage to a subset of the terminology (Table 8). The complete SNOMED CT terminology was used in 64% (49/76) of the publications. A subset of the terminology was used in 30% (23/76). The size of these subsets could vary from less than 10 concepts [47] to several thousand [37].

**Table 8:** Subset of Systematized Nomenclature of Medicine Clinical Terms used in publications.

Subset of SNOMED CT^a^ used	Publications (N=76), n (%)
Full terminology	49 (64)
Subset	23 (30)
Not available	4 (5)

^a^SNOMED CT: Systematized Nomenclature of Medicine Clinical Terms.

### Advanced Functionalities Used

SNOMED CT includes a large set of functionalities atop the classical ontology usage, among which the most interesting are the combinatorial possibilities that offer postcoordination. Table 9 shows whether a publication performed postcoordination to a certain extent. Among the 13 publications using this feature, 4 of them (5%) [30,35,36,49], all by the same first author, specifically mentioned the compositional grammar published by SNOMED CT [10]; however, the others do not elaborate nor propose simple postcoordination such as combining concepts with a “+” sign.

**Table 9:** Use of postcoordination.

Usage of postcoordination	Publications (N=76), n (%)
No	61 (80)
Yes	13 (17)
Not available	2 (3)

## Discussion

### Principal Findings

SNOMED CT is mostly used to represent information found in the complementary exam reports (18/796, 24%). This is potentially influenced by an important number of studies focusing on radiology [119] and pathology, as complementary exam reports are often produced by those divisions. Moreover, pathology being historically the field of SNOMED CT, it could have influenced its application in this domain. In addition, these types of reports are usually focused on specific clinical questions and arguably convey more specific informational content.

The second type of free text represented in our results is narrative notes (16/76, 21%). Potentially, this can be explained by the large conceptual span of SNOMED CT, which allows good informational coverage on textual data.

Finally, a large set of articles do not filter data for specific types. This is explained by publications focusing more on providing a solution to map SNOMED CT concepts to text in general, without targeting a specific type of document. This is supported by the fact that those publications have the mapping to SNOMED CT concepts as their final goal in 9 out of 14 (64%) publications, which is significantly higher than the rest of the selection (16/76, 21%).

In the selection, only 7 out of 76 (9%) publications focused on a language other than English. Multiple reasons can explain this predominance of the English language in research studies. First, NLP is known to be dependent on language. Work performed in a language cannot easily be transferred to other languages. Therefore, the overhead to begin NLP research in another language is substantial and brings few rewards in the first stages, as the breakthrough has already been published in another language.

Second, SNOMED CT—like most international classifications and ontologies—was first published only in English. Rule-based methods, which are the most frequently used methods for SNOMED CT mapping, rely on the assumption that the description of a concept can be directly mapped to free text, which is not possible when the language of the text is not the language of the classification. However, translations of SNOMED CT exist for Spanish, Swedish, and recently French [120]. Therefore, there is hope for new developments as the barriers to the language start to be overcome.

Finally, several publications use public data sets such as the i2b2-shared task data sets [33,34,41,50,51] or the MIMIC II [52] data set as the sources of narrative documents. These public data sets are valuable for promoting research in NLP on clinical free text and are the subject of many publications. The availability of such resources in languages other than English is scarce.

Unsurprisingly, the most frequent reason for mapping to SNOMED CT is information extraction (44/76, 39%), as the ability of SNOMED CT to represent medical knowledge is the core feature of this terminology. Nonetheless, 26 articles (34.21%) used the resulting mapping as a feature in a classification task, usually using a learning algorithm such as support vector machines or conditional random fields [53,54]. SNOMED CT is used in these cases as a proxy for the semantic content of the data, between free text and structured data, to simplify the task of classification and improve results.

Similarity evaluation is the goal in 4 publications (5.26%). Whether it is to compare cases [55], documents [29,37], or concepts [56], the similarity is computed using the SNOMED CT concepts. Both the polyhierarchy and the defining relationships can be used to compute the semantic distance between concepts. However, only 3 of the publications used them. This is an example of the added value SNOMED CT can bring to the secondary use of medical data.

Only 21% (16/76) of the publications mapped free text to SNOMED CT as a final objective. This is explained by the large number of publications reusing a mapping tool developed in a previous publication for new goals. To illustrate this phenomenon, Nguyen et al [38] reuse the software Medtex presented their study in multiple publications [14,30,35,36,49]. This is also true for large publicly available tools such as MCVS [16,17,57] or MTERMS [41,42].

The 3 most represented software in the selection—Medtex, MTERMS, and MCVS—are not available for public use. They mainly appear in publications by teams that have developed them. However, 2 software packages are available under an open-source license and can be freely used to map free text to SNOMED CT concepts, Metamap (and MMTx), and cTAKES. These tools are available to perform automatic annotation with SNOMED CT; however, none of them are specifically aimed at this ontology nor do they include features such as postcoordination or multiple language support. There is currently no clear solution for mapping free text to SNOMED CT concepts out of the box with a specific focus on this ontology and its features. This could explain the overall small number of publications in the selection.

Rule-based methods are largely used to perform mapping (53/76, 70%). This tends to show that they are more suited for this task. This phenomenon could be due to the large number of concepts in SNOMED CT. The amount of annotated data needed to automatically map free text with more than 340,000 classes is enormous and would require an important investment.

The evaluations of the automatic mapping found in publications show that this is not a trivial task. Most solutions for mapping lack a clear and definitive evaluation, and when available, they usually focus on a small set of documents; they use a subset of the terminology or do not rely on a gold standard. This gap in research could be explained by several reasons.

The number of concepts in SNOMED CT is large, and all granularities coexist. To express a simple concept such as *Tuberculous pneumonia*, a single concept can be used: 80003002 (Tuberculous pneumonia [disorder]) or any combination of less granular concepts (233604007 | Pneumonia [disorder], 233618000 |Mycobacterial pneumonia [disorder], 56717001 | Tuberculosis [disorder], 113858008 |Mycobacterium tuberculosis complex [organism], etc). However, all these representations can be equally correct from a semantic point of view. Therefore, it is difficult to compute the recall as a gold standard, which usually represents only one of these representations. Moreover, SNOMED CT contains 18 subhierarchies focusing on different thematics (clinical findings, body structure, etc), which make the decision of which concept to use even more difficult. For example, the hierarchy of the observable entities defines what can be observed in a patient, but the clinical finding hierarchy contains the results of those observations. The choice between a finding and an observable entity is not always clear and can heavily depend on the context. Finally, the usage of postcoordinated terms increases the set of expressions that can be used to represent the same concept. Overall, the task of evaluating the automatic mapping of natural language to a SNOMED CT concept lacks a pragmatic and applicable method; therefore, it is often limited to small-scale evaluations or manual validations.

The version of SNOMED used in publications (SNOMED, SNOMED CT, or SNOMED RT) is not always specified, especially when the usage of this terminology is not the main goal of the research. Moreover, the usage of SNOMED CT is implicit when UMLS is used. This remark, as well as the small number of publications mentioning postcoordination, emphasizes the fact that SNOMED CT is often seen as a simple terminology, without the need to use its advanced features. This phenomenon is also shown by the fact that only a subset of the terminology is used in 64% (49/76) of the publications. Using a subset simplifies the mapping task by reducing complexity but also prevents from benefiting from the power of the polyhierarchy and the relationships among concepts.

As clinical free text is written in natural language and since SNOMED CT is designed as a formal language, it is surprising that very few papers use this functionality when mapping to free text. Although this can be explained by the fact that even if SNOMED International provides compositional grammar, there is, to the best of our knowledge, no explicit roadmap to use it for such a task. Postcoordination requires deep knowledge of the terminology and access to a terminology server that handles the resulting data. As SNOMED International is not a software provider, this has to be achieved either using the open-source server Snowstorm [121], for which SNOMED International does not provide technical support, or by relying on a private company software.

This work shows that although SNOMED CT is widely used in health care, its use to represent free-text data still remains a challenge. Polyhierarchy and compositional grammar are at the core of SNOMED CT and they can bring significant value to data; however, when it comes to mapping concepts to free text, there seems to be a margin for approaches that take advantage of those features. The same can be observed on the usage of SNOMED CT to process free text in languages other than English.

Although machine learning is clearly on the rise in multiple fields of medical informatics and scientific research in general, it is rarely used to map free text to SNOMED CT, most probably because of the size of the corpus needed to train on such a large set of classes. In contrast, rule-based symbolic approaches seem more suited and are used to map large terminologies to free-text data. A combination of the strengths of both hybrid approaches could be a way to improve performance.

Finally, an openly available tool that would process free texts and map them to SNOMED CT concepts is yet to be created.

### Limitations

Although the review has been conducted following a systematic approach, this work has some limitations.

The last publication research was conducted in August 2019. It is possible that new publications have been published since then. As we have observed, the number of publications selected per year is reducing; therefore, we consider the impact of this gap to be arguably small. Although the recursive reference review has been performed with the aim of broadening the scope of the included papers, it is possible that some studies that have not yet been cited by other papers have not been considered. For example, the high-throughput phenotyping NLP system described by Schlegel et al. [122] did not appear in the search nor during the recursive reference review. This system uses a series of linguistic and semantic indexes to process clinical data and characterizes it using ontologies such as SNOMED CT and the International Classification of Diseases 10.

In the selection, a large number of publications are published by the same groups of authors and propose similar works. This could result in an overestimation of the impact of those publications on a complete selection.

Finally, it is possible that because of the choice to focus on biomedical databases to gather publications, some articles published on more engineering-oriented databases have not been included.

### Conclusions

In conclusion, clinical free-text processing and SNOMED CT have been an important subject for research, but the number of publications has been diminishing in recent years. Most of the publications that we found mapped free text to SNOMED CT to obtain a semantic representation of the data and used it as a first step toward other goals such as document classification or information retrieval.

Almost none of the publications used advanced features of SNOMED CT, such as the polyhierarchy or postcoordination. Most publications conceive SNOMED CT only as a terminology, a dictionary, or a resource for synonyms.

Publications focusing on languages other than English are rare and, if software exists for mapping English free text to SNOMED CT, most of them are not available for public use or focus on UMLS and not strictly on SNOMED CT. There is currently no easy solution for mapping free-text data into the SNOMED CT concepts, especially if the source language is different from English or if postcoordination is needed.

However, the need for formal semantic representation of health care data and the secondary use of free-text data is high, and automatic encoding into a compositional ontology could be a way to achieve interoperability.

## Appendix

Multimedia Appendix 1 List of 76 articles included in the review process.

## Abbreviations

cTAKES: clinical Text Analysis and Knowledge Extraction System
CUI: concept unique identifier
MCVS: Mayo Clinic Vocabulary Server
MMTx: Metamap transfer
MTERMS: medical Text Extraction Reasoning and Mapping System
NLM: National Library of Medicine
NLP: natural language processing
OBA: Open Biomedical Annotator
RapTAT: Rapid Text Annotation Tool
SNOMED CT: Systematized Nomenclature of Medicine Clinical Terms
SNOMED RT: SNOMED reference terminology
UMLS: Unified Medical Language System
