# Supplementary material for: Use of the Systematized Nomenclature of Medicine Clinical Terms (SNOMED CT) for Processing Free Text in Health Care: Systematic Scoping Review
Source: J Med Internet Res. 2021 Jan 26;23(1):e24594. doi: 10.2196/24594 (PMC7872838; doi:10.2196/24594)
Supplement: Multimedia Appendix 1 [file jmir_v23i1e24594_app1.pdf]

# Multimedia appendix 1: List of 76 references selected in the review

| First Author | Publication Year | Title | Journal |
|---|---|---|---|
| **Burkhart, Lisa** | 2005 | Mapping parish nurse documentation into the nursing interventions classification: A research method | CIN - Computers Informatics Nursing |
| **Elkin, Peter L.** | 2005 | A controlled trial of automated classification of negation from clinical notes | BMC Medical Informatics and Decision Making |
| **Rindflesch, Thomas C** | 2005 | Medical Facts to Support Inferencing in Natural Language Processing | AMIA Annual Symposium proceedings |
| **Long, William** | 2005 | Extracting diagnoses from discharge summaries. | AMIA Annual Symposium proceedings |
| **Pakhomov, Serguei V** | 2005 | High throughput modularized NLP system for clinical text | Proceedings of the ACL 2005 on Interactive poster and demonstration sessions - ACL '05 |
| **Shah, Nigam H** | 2006 | Ontology-based annotation and query of tissue microarray data. | AMIA Annual Symposium proceedings |
| **Elkin, Peter L.** | 2006 | Evaluation of the content coverage of SNOMED CT: Ability of SNOMED clinical terms to represent clinical problem lists | Mayo Clinic Proceedings |
| **Melton, Genevieve B.** | 2006 | Inter-patient distance metrics using SNOMED CT defining relationships | Journal of Biomedical Informatics |
| **Brown, Steven H** | 2006 | eQuality: Electronic quality assessment from narrative clinical reports | Mayo Clinic Proceedings |
| **Meystre, Stéphane** | 2006 | Natural language processing to extract medical problems from electronic clinical documents: Performance evaluation | Journal of Biomedical Informatics |
| **Patrick, Jon D.** | 2007 | An automated system for conversion of clinical notes into SNOMED clinical terminology | Conferences in Research and Practice in Information Technology Series |
| **Ogren, Philip V.** | 2007 | Constructing evaluation corpora for automated clinical named entity recognition | Medinfo 2007: Proceedings of the 12th World Congress on Health (Medical) Informatics; Building Sustainable Health Systems |
| **Brown, Steven H** | 2008 | eQuality for all: Extending automated quality measurement of free text clinical narratives. | AMIA Annual Symposium proceedings |
| **Johnson, Stephen B.** | 2008 | An Electronic Health Record Based on Structured Narrative | Journal of the American Medical Informatics Association |
| **Patrick, Jon D.** | 2008 | Developing SNOMED CT subsets from clinical notes for intensive care service | Health Care and Informatics Review Online |
| **Elkin, Peter L.** | 2008 | NLP-based identification of pneumonia cases from free-text radiological reports. | AMIA Annual Symposium proceedings |
| **Ryan, Angela** | 2008 | Introduction of Enhancement Technologies into the Intensive Care | Health Information Management Journal |
| **Ruch, Patrick** | 2008 | Automatic medical encoding with SNOMED categories. | BMC Medical Informatics and Decision Making |
| **Li, Dingcheng** | 2008 | Conditional Random Fields and Support Vector Machines for Disorder Named Entity Recognition in Clinical Texts | Proceedings of the workshop on current trends in biomedical |

| | | | |
|---|---|---|---|
| | | | natural language processing |
| **Kipper-Schuler, Karin C.** | 2008 | System evaluation on a named entity corpus from clinical notes | Language resources and evaluation conference, LREC |
| **Savova, Guergana K** | 2008 | UIMA-based Clinical Information Extraction System | Towards enhanced interoperability for large HLT systems: UIMA for NLP |
| **Shah, Nigam H** | 2009 | Ontology-driven indexing of public datasets for translational bioinformatics | BMC Bioinformatics |
| **Aseervatham, Sujeevan** | 2009 | Semi-structured document categorization with a semantic kernel | Pattern Recognition |
| **Matheny, Michael E.** | 2009 | Detection of blood culture bacterial contamination using natural language processing. | AMIA Annual Symposium proceedings |
| **Wang, Yefeng** | 2009 | Annotating and recognising named entities in clinical notes | Proceedings of the ACL-IJCNLP 2009 Student Research Workshop |
| **Wang, Yefeng** | 2009 | Cascading Classifiers for Named Entity Recognition in Clinical Notes | Proceedings of the Workshop on Biomedical Information Extraction |
| **Nguyen, Anthony N** | 2009 | A simple pipeline application for identifying and negating SNOMED clinical terminology in free text | HIC 2009: Proceedings |
| **Nguyen, Anthony N.** | 2010 | Symbolic rule-based classification of lung cancer stages from free-text pathology reports | Journal of the American Medical Informatics Association |
| **Arnot-Smith, J.** | 2010 | Patient safety incidents involving neuromuscular blockade: Analysis of the UK National Reporting and Learning System data from 2006 to 2008 | Anaesthesia |
| **Savova, Guergana K.** | 2010 | Mayo clinical Text Analysis and Knowledge Extraction System (cTAKES): Architecture, component evaluation and applications | Journal of the American Medical Informatics Association |
| **Přečková, Petra** | 2010 | Language of Czech Medical Reports and Classification Systems in Medicine | European Journal for Biomedical Informatics |
| **Lee, Dennis H.** | 2010 | A method for encoding clinical datasets with SNOMED CT | BMC Medical Informatics and Decision Making |
| **Patrick, Jon D.** | 2010 | High accuracy information extraction of medication information from clinical notes: 2009 i2b2 medication extraction challenge | Journal of the American Medical Informatics Association |
| **Wagholikar, Amol S** | 2011 | Identifying symptom groups from Emergency Department presenting complaint free text using SNOMED CT. | AMIA Annual Symposium proceedings |
| **Nguyen, Anthony N.** | 2011 | Automatic extraction of cancer characteristics from free-text pathology reports for cancer notifications | Studies in Health Technology and Informatics |
| **Fung, Kin Wah** | 2011 | Testing Three Problem List Terminologies in a simulated data entry environment. | AMIA Annual Symposium proceedings |
| **So, Eun Young** | 2011 | Mapping medical records of gastrectomy patients to SNOMED CT | Studies in Health Technology and Informatics |
| **Martinez, David** | 2011 | Information extraction from pathology reports in a hospital setting | Proceedings of the 20th ACM international conference on Information and knowledge management - CIKM '11 |

| | | | |
|---|---|---|---|
| **Murff, Harvey J.** | 2011 | Automated identification of postoperative complications within an electronic medical record using natural language processing | Journal of the American Medical Informatics Association |
| **Zhou, Li** | 2011 | Using Medical Text Extraction, Reasoning and Mapping System (MTERMS) to process medication information in outpatient clinical notes. | AMIA Annual Symposium proceedings |
| **Warden, Graham I** | 2011 | Leveraging terminologies for retrieval of radiology reports with critical imaging findings. | AMIA Annual Symposium proceedings |
| **Patrick, Jon D.** | 2011 | A knowledge discovery and reuse pipeline for information extraction in clinical notes | Journal of the American Medical Informatics Association |
| **Matheny, Michael E.** | 2012 | Detection of infectious symptoms from VA emergency department and primary care clinical documentation | International Journal of Medical Informatics |
| **Liu, Hongfang** | 2012 | Using SNOMED-CT to encode summary level data - a corpus analysis. | AMIA Joint Summits on Translational Science proceedings |
| **Přečková, Petra** | 2012 | Measuring diversity in medical reports based on categorized attributes and international classification systems. | BMC medical informatics and decision making |
| **Nguyen, Anthony N.** | 2012 | Classification of pathology reports for Cancer Registry notifications | Studies in Health Technology and Informatics |
| **Nguyen, Anthony N.** | 2012 | Structured pathology reporting for cancer from free text: Lung cancer case study | Electronic Journal of Health Informatics |
| **Pivovarov, Rimma** | 2012 | A hybrid knowledge-based and data-driven approach to identifying semantically similar concepts | Journal of Biomedical Informatics |
| **Davis, Kailah** | 2012 | Identification of pneumonia and influenza deaths using the death certificate pipeline | BMC Medical Informatics and Decision Making |
| **ul Muntaha, Sidrat** | 2012 | Entity recognition of pharmaceutical drugs in Swedish clinical text | Proceedings of SLTC 2012 The Fourth Swedish Language Technology Conference |
| **Skeppstedt, Maria** | 2012 | Rule-based Entity Recognition and Coverage of SNOMED CT in Swedish Clinical Text. | Proceedings of the eight international conference on language resources and evaluation (LREC'12) |
| **Zuccon, Guido** | 2013 | Automatic Classification of Free-Text Radiology Reports to Identify Limb Fractures using Machine Learning and the SNOMED CT Ontology. | AMIA Joint Summits on Translational Science proceedings |
| **Butt, Luke** | 2013 | Classification of cancer-related death certificates using machine learning | Australasian Medical Journal |
| **Jindal, Prateek** | 2013 | Extraction of events and temporal expressions from clinical narratives | Journal of Biomedical Informatics |
| **So, Eun Young** | 2013 | Exploring the possibility of information sharing between the medical and nursing domains by mapping medical records to SNOMED CT and ICNP | Healthcare Informatics Research |
| **Mabotuwana, Thusitha** | 2013 | An ontology-based similarity measure for biomedical data - Application to radiology reports | Journal of Biomedical Informatics |
| **Hong, Yi** | 2013 | Content analysis of reporting templates and free-text radiology reports | Journal of Digital Imaging |

| | | | |
|---|---|---|---|
| **Henriksson, Aron** | 2013 | Identifying synonymy between SNOMED clinical terms of varying length using distributional analysis of electronic health records | AMIA Annual Symposium proceedings |
| **Gobbel, Glenn T.** | 2014 | Development and evaluation of RapTAT: A machine learning system for concept mapping of phrases from medical narratives | Journal of Biomedical Informatics |
| **Zhou, Li** | 2014 | Representation of Information about Family Relatives as Structured Data in Electronic Health Records | Applied Clinical Informatics |
| **Goss, Foster R.** | 2014 | An evaluation of a natural language processing tool for identifying and encoding allergy information in emergency department clinical notes. | AMIA Annual Symposium proceedings |
| **Ou, Ying** | 2014 | Automatic structured reporting from narrative cancer pathology reports | Electronic Journal of Health Informatics |
| **Skeppstedt, Maria** | 2014 | Automatic recognition of disorders, findings, pharmaceuticals and body structures from clinical text: An annotation and machine learning study | Journal of Biomedical Informatics |
| **Koopman, Bevan** | 2015 | Automatic ICD-10 classification of cancers from free-text death certificates | International Journal of Medical Informatics |
| **Koopman, Bevan** | 2015 | Automatic classification of diseases from free-text death certificates for real-time surveillance | BMC medical informatics and decision making |
| **Nguyen, Anthony N.** | 2015 | Assessing the Utility of Automatic Cancer Registry Notifications Data Extraction from Free-Text Pathology Reports. | AMIA Annual Symposium proceedings |
| **Plasek, Joseph M.** | 2016 | Food entries in a large allergy data repository | Journal of the American Medical Informatics Association |
| **Nguyen, Anthony N.** | 2016 | Automated cancer registry notifications: validation of a medical text analytics system for identifying patients with cancer from a state-wide pathology repository | AMIA Annual Symposium proceedings |
| **Lin, Chin** | 2017 | Artificial Intelligence Learning Semantics via External Resources for Classifying Diagnosis Codes in Discharge Notes | Journal of Medical Internet Research |
| **Zhang, Rui** | 2017 | Enriching the international clinical nomenclature with Chinese daily used synonyms and concept recognition in physician notes | Bmc Medical Informatics and Decision Making |
| **Zvara, Karel** | 2017 | Tool-supported Interactive Correction and Semantic Annotation of Narrative Clinical Reports | Methods of Information in Medicine |
| **Nguyen, Anthony N.** | 2018 | Computer-Assisted Diagnostic Coding: Effectiveness of an NLP-based approach using SNOMED CT to ICD-10 mappings | AMIA Annual Symposium proceedings |
| **Koopman, Bevan** | 2018 | Extracting cancer mortality statistics from death certificates: A hybrid machine learning and rule-based approach for common and rare cancers | Artificial Intelligence in Medicine |
| **Mujtaba, Ghulam** | 2018 | Classification of forensic autopsy reports through conceptual graph-based document representation model | Journal of Biomedical Informatics |
| **Jackson, Richard** | 2018 | Knowledge discovery for Deep Phenotyping serious mental illness from Electronic Mental Health records | F1000Research |

| | | | |
|---|---|---|---|
| **Tahmasebi, Amir M.** | 2019 | Automatic Normalization of Anatomical Phrases in Radiology Reports Using Unsupervised Learning | Journal of Digital Imaging |
